# Myocardial tissue and metabolism characterization in men with alcohol consumption by cardiovascular magnetic resonance and 11C-acetate PET/CT

**DOI:** 10.1186/s12968-020-00614-2

**Published:** 2020-04-16

**Authors:** Shuai Liu, Xue Lin, Ximin Shi, Ligang Fang, Li Huo, Fei Shang, Juhani Knuuti, Chunlei Han, Xiaomeng Wu, Rui Guo, Haiyan Ding, Runhua Zhang, Huimin Duan, Jie Ding, Haiqun Xing, Xihai Zhao

**Affiliations:** 1 Center for Biomedical Imaging Research, Department of Biomedical Engineering, Tsinghua University School of Medicine, Haidian District, Beijing, 100084 China; 2 Department of Cardiology, Peking Union Medical College Hospital, Chinese Academy of Medical Science, Beijing, China; 3 Department of Nuclear Medicine, Peking Union Medical College Hospital, Chinese Academy of Medical Science, 1# Shuaifuyuan, Dongcheng District, Beijing, 100730 China; 4 Beijing Key Laboratory of Molecular Targeted Diagnosis and Therapy in Nuclear Medicine, Beijing, China; 5 Department of Biomedical Engineering, Beijing Institute of Technology School of Life Science, Beijing, China; 6 Turku PET Center, Turku University Hospital and University of Turku, Turku, Finland; 7 Department of Neurology, Beijing Tiantan Hospital, Capital Medical University, Beijing, China; 8 Department of Medical Engineering, First Affiliated Hospital of PLA General Hospital, Beijing, China

**Keywords:** Alcoholic cardiomyopathy, T1 mapping, Magnetic resonance imaging, Oxidative metabolism, Positron emission tomography

## Abstract

**Background:**

Chronic alcohol consumption initially leads to asymptomatic left ventricular dysfunction, but can result in myocardial impairment and heart failure if ongoing. This study sought to characterize myocardial tissues and oxidative metabolism in asymptomatic subjects with chronic alcohol consumption by quantitative cardiovascular magnetic resonance (CMR) and 11C-acetate positron emission tomography (PET)/computed tomography (CT).

**Methods:**

Thirty-four male subjects (48.8 ± 9.1 years) with alcohol consumption > 28 g/day for > 10 years and 35 age-matched healthy male subjects (49.5 ± 9.7 years) underwent CMR and 11C-acetate PET/CT. Native and post T1 values and extracellular volume (ECV) from CMR and Kmono and K1 from PET imaging were measured. Quantitative measurements by CMR and PET imaging were compared between subjects with moderate to heavy alcohol consumption and healthy controls, and their correlations were also analyzed.

**Results:**

Compared to healthy controls, subjects with alcohol consumption showed significantly shorter native T1 (1133 ± 65 ms vs. 1186 ± 31 ms, *p* < 0.001) and post T1 (477 ± 42 ms vs. 501 ± 38 ms, *p* = 0.008) values, greater ECV (28.2 ± 2.2% vs. 26.9 ± 1.3%, *p* = 0.003), marginally lower Kmono (57.6 ± 12.1 min^− 1^ × 10^− 3^ vs. 63.0 ± 11.7 min^− 1^ × 10^− 3^, *p* = 0.055), and similar K1 (0.82 ± 0.13 min^− 1^ vs. 0.83 ± 0.15 min^− 1^, *p* = 0.548) after adjusting for confounding factors. There were no significant differences in CMR measurements and K1 between subjects with heavy and moderate alcohol consumption (all *p* > 0.05). In contrast, subjects with heavy alcohol consumption showed significantly lower Kmono values compared to those with moderate alcohol consumption (52.9 ± 12.1 min^− 1^ × 10^− 3^ vs. 63.7 ± 9.2 min^− 1^ × 10^− 3^, *p* = 0.012). Strong and moderate correlations were found between K1 and ECV in healthy controls (*r* = 0.689, *p* = 0.013) and subjects with moderate alcohol consumption (*r* = 0.518, *p* = 0.048), respectively.

**Conclusion:**

Asymptomatic men with heavy alcohol consumption have detectable structural and metabolic changes in myocardium on CMR and 11C-acetate PET/CT. Compared with quantitative CMR, 11C-acetate PET/CT imaging may be more sensitive for detecting differences in myocardial damage among subjects with moderate to heavy alcohol consumption.

## Introduction

Alcohol consumption is the third leading lifestyle-related cause of death for people in the US, behind tobacco and improper diet/lack of physical activity, and is responsible for 3.8% of all deaths globally [1–3]. Alcoholic cardiomyopathy accounts for up to 40% of patients with non-ischemic dilated cardiomyopathy [4]. Without complete abstinence, the 4-year mortality rate for alcohol-induced dilated cardiomyopathy approaches 50% [5]. Although moderate alcohol consumption may not be harmful to the cardiovascular system, excessive intake of alcohol can damage the myocardium via stimulating the apoptosis of cardiomyocytes and inhibiting energy metabolism [6–9]. Initially, alcohol consumption leads to asymptomatic left ventricular (LV) dysfunction, and can result in symptoms of heart failure if continues. Therefore, early assessment of the changes in structure and metabolism of myocardial tissues in subjects with alcoholism at asymptomatic stage is important for prevention of irreversible outcomes, such as dilated cardiomyopathy and heart failure.

Quantitative cardiovascular magnetic resonance (CMR) imaging, such as T1 mapping, can measure the native value of longitudinal (spin-lattice) relaxation time (T1) of myocardial tissues and extracellular volume (ECV), which represents the size of the extracellular space and reflects interstitial diseases [10]. The native T1 values of myocardial tissues vary with myocardial extracellular water (edema), focal or diffuse fibrosis, fat, iron, and amyloid proteins. Meanwhile, quantitative parameters measured from 11C-acetate positron emission tomography (PET) imaging, such as Kmono and K1, have been largely utilized to characterize myocardial metabolism in heart diseases [11–14]. Kmono is considered as a robust marker for evaluation of myocardial oxygen consumption (MVO_2_), while K1 is correlated with myocardial blood flow (MBF) in absolute terms. As such, it is possible to assess changes in structure and metabolism of myocardial tissues in subjects with alcohol consumption by combining quantitative CMR with 11C-acetate PET/computed tomography (CT) imaging.

This study sought to investigate tissue characterization and metabolic disorders in asymptomatic subjects with chronic alcohol consumption by using quantitative CMR and 11C-acetate PET/CT imaging.

## Methods

### Study sample

Male adults with a history of drinking > 10 years and average alcohol consumption >2std/day (1std/day = 14 g pure ethanol) were recruited. Subjects with one of the following conditions were excluded: 1) hypertension; 2) diabetes mellitus; 3) coronary artery disease; 4) LV ejection fraction (LVEF) < 50% or abnormalities in cardiac structure on ultrasound imaging; 5) congenital heart disease; 6) renal dysfunction (glomerular filtration rate < 60 mL/min); 7) atrial fibrillation or premature beat on electrocardiogram; 8) cirrhosis; and 9) contraindications to CMR examination. Healthy male adults with matched ages but without history of alcohol consumption or with only occasional alcohol intake (< 100 g/month) were enrolled as healthy controls. The following demographic and clinical information was collected: age, height, weight, blood pressure, heartbeat, and history of smoking (current or one-time smoker). A questionnaire was conducted to collect the following information on alcohol consumption: 1) how many times do you drink in a week? 2) which type of alcoholic beverage do you drink, e.g. beer, wine or liquor? 3) how much of alcoholic beverage do you drink per drinks? 4) how many years have you maintained this type of drinking? The study protocol was approved by Institutional Review Board of Tsinghua University School of Medicine and a signed consent form was obtained from each subject prior to enrollment.

#### CMR imaging

CMR imaging was performed on a 3 Tesla scanner (Achieva TX, Philips Healthcare, Best, The Netherlands) with a 32-channel cardiac coil. The CMR protocol included CINE, pre- and post-contrast enhanced T1 mapping. The image parameters were as follows: CINE: turbo field echo; repetition time/echo time 3.2/1.6 ms, field of view 320 × 320 mm^2^, flip angle 40°, and slice thickness 8 mm; and T1-mapping: 3–3-5 modified Look-Locker inversion recovery (MOLLI), repetition time/echo time 2.3/0.9 ms, field of view 320 × 320 mm^2^, flip angle 35°, and slice thickness 8 mm. For CINE imaging, multiple slices were acquired on short-axis view. T1 mapping was acquired on three short-axis views of the mid ventricular septum and 10 mm up and down with breath holding. Post contrast enhanced T1-mapping was conducted 10 min after administration of a Gadolinium-based contrast agent (Magnevist, Bayer Schering Pharma, Berlin, Germany) with a dose of 0.15 mmol/kg and a flow rate of 2 ml/s.

#### PET/CT imaging

PET/CT imaging was performed on a hybrid PET/CT scanner (PoleStar m660, Sinounion, Healthcare Inc., Beijing, China). After a low dose CT scanning for location (120 kV and 140 mA), 11C-acetate with a dose of 740 MBq was injected intravenously, followed by a 40-min dynamic PET scan. A total of 53 frames (15 × 10 s, 15 × 30 s, 16 × 60 s, and 7 × 120 s) were reconstructed using the 3D OSEM+TOF algorithm on a Precision workstation (Sinounion, Healthcare Inc., Beijing, China) with an object space of 192 × 192 × 117 and a voxel size of 3.15 × 3.15 × 1.87 mm^3^ after correction for dead time, decay and measured photon attenuation.

### Data analysis

CMR images were analyzed with group-wise registration using the open source toolbox Elastix and non-linear curve fitting based on the Levenberg-Marquardt algorithm was then performed. Two radiologists who had > 5 years’ experience in CMR measured the values for LV and right ventricular (RV) end diastolic volumes (LVEDV and RVEDV), left and right ventricular end systolic volumes (LVESV and RVESV), LV and RV stroke volumes (LVSV and RVSV), LVEF, RV ejection fraction (RVEF), cardiac output (CO), and LV mass using a CMR workstation (Philips Extended MR WorkSpace 2.6.3.4). The values of native T1, post T1 and ECV at the middle interventricular septum on three slices were measured by two radiologists. The mean values of native T1, post T1 and ECV on three slices were then recorded. The radiologists were blinded to history of alcohol consumption and PET/CT imaging data.

PET/CT images were reviewed by two reviewers with > 10 years` experience in nuclear medicine who were blinded to history of alcohol consumption and CMR imaging data. Kmono was calculated by mono-exponential fitting and K1 was calculated using a single-compartmental model with LV time-activity curve as the input function. Both Kmono and K1 were calculated at two levels: global for the entire myocardium and segmented for the 17-segment model [15]. All analyses were performed using the Carimas software (v2.9, developed in Turku PET Centre of Finland, http://www.turkupetcentre.fi/carimas). A total of 20 subjects with alcohol consumption were randomly selected for testing the reproducibility of Kmono and K1 measurements. Each observer reviewed PET/CT images using Carimas independently, then performed reorientation, short axis definition and manual correction. One reviewer analyzed the data of PET/CT again after 1 month to minimize memory bias.

### Statistical analysis

Continuous variables were presented as mean ± standard deviation (SD). Kmono and K1 measurements in the septal segments (Seg8 and Seg9), corresponding to the same regions analyzed in CMR, were taken. The mean values for native T1, post T1, ECV, Kmono and K1 from the two reviewers were compared between alcohol consumption groups and healthy controls using an independent *t* test. The linear relationship between measurements from CMR and PET imaging was determined using Spearman’s correlation coefficient. For testing reproducibility, intraclass correlation coefficient (ICC) and a corresponding 95% confidence interval (CI) were calculated to determine inter- and intra-observer agreement in measuring Kmono and K1. A *p* < 0.05 was considered statistically significant and all statistical analysis was conducted with SPSS (v 25.0 Statistical Package for the Social Sciences, International Business Machines, Inc., Armonk, New York, USA).

## Results

In total, 40 subjects with moderate to heavy alcohol consumption and 38 healthy controls were recruited for this study between February and December 2017. Six subjects with moderate to heavy alcohol consumption and 3 healthy controls were excluded due to the following reasons: 1) failure of contrast agent injection for CMR imaging (*n* = 1) or tracer for PET/CT scanning (*n* = 2); 2) poor image quality for CMR imaging (*n* = 5); and 3) claustrophobia (n = 1). Of the remaining 35 healthy controls, only the first 12 (34.3%) underwent PET imaging. Subjects with a history of alcohol consumption were divided into moderate (<5std/day, *n* = 15) and heavy consumption groups (≥5std/day, *n* = 19), according to the 2015 to 2020 Dietary Guidelines for Americans 8th Edition (mild consumption: <2std/day; moderate consumption: 2-5std/day; heavy consumption: ≥5std/day. 1std/day = 14 g pure ethanol). Clinical characteristics and measurements from echocardiography are shown in Table 1. All subjects with moderate to heavy alcohol consumption and healthy controls were male with mean ages of 48.8 ± 9.1 and 49.5 ± 9.7 years (*p* = 0.771), respectively. Compared to healthy controls, subjects with moderate to heavy alcohol consumption exhibited significantly higher diastolic blood pressure (77.6 ± 8.3 mmHg vs. 73.2 ± 6.7 mmHg, *p* = 0.020), prevalence of smoking (62.0% vs. 20.0%, *p* < 0.001), body mass index (BMI) (26.0 ± 2.9 kg/m^2^ vs. 23.2 ± 2.2 kg/m^2^, *p* < 0.001), LVEDV (116.2 ± 21.5 ml vs. 106.0 ± 20.2 ml, *p* = 0.046), LVESV (41.7 ± 12.2 ml vs. 35.3 ± 7.6 ml, *p* = 0.010), RVEDV (130.3 ± 24.5 ml vs. 117.5 ± 26.3 ml, *p* = 0.041), and RVESV (64.3 ± 15.0 ml vs. 54.6 ± 13.2 ml, *p* = 0.006). No significant differences were found in systolic blood pressure, LVEF, RVEF, LVSV, RVSV, CO and LV mass (all *p* > 0.05) between subjects with moderate to heavy alcohol consumption and healthy controls. No significant differences were observed in clinical characteristics between subjects with heavy and moderate alcohol consumption (all p > 0.05).

**Table 1:** Clinical characteristics of the study population

	Mean ± SD or n (%)				P^a^	P^b^
	Subjects with moderate to heavy alcohol consumption			Healthy controls (*n* = 35)		
	All (*n* = 34)	Heavy consumption (*n* = 19)	Moderate consumption (*n* = 15)			
Age, years	48.8 ± 9.1	49.9 ± 11.1	47.4 ± 5.9	49.5 ± 9.7	0.771	0.407
BMI, kg/m^2^	26.0 ± 2.9	25.8 ± 3.1	26.3 ± 2.8	23.2 ± 2.2	< 0.001	0.672
Smoke	21 (62.0)	12 (63.2)	9 (60.0)	7 (20.0)	< 0.001	0.856
SBP, mmHg	120.0 ± 11.2	116.8 ± 11.4	124.1 ± 9.8	116.5 ± 10.8	0.192	0.055
DBP, mmHg	77.6 ± 8.3	76.5 ± 8.3	78.9 ± 8.5	73.2 ± 6.7	0.020	0.424
Alcohol consumption						
Duration, years	26.2 ± 8.9	26.5 ± 10.7	25.8 ± 6.3	0	–	0.806
Dose, g/day	115.8 ± 119.7	167.2 ± 140.6	50.5 ± 13.2	0	–	0.002
Left ventricle						
LVEDV, mL	116.2 ± 21.5	115.4 ± 25.4	117.2 ± 16.0	106.0 ± 20.2	0.046	0.808
LVESV, mL	41.7 ± 12.2	43.2 ± 13.5	39.8 ± 10.5	35.3 ± 7.6	0.010	0.425
LVSV, mL	74.4 ± 17.7	72.1 ± 18.5	77.4 ± 16.7	70.7 ± 16.7	0.369	0.396
LVEF, %	64.9 ± 6.7	63.8 ± 6.1	66.3 ± 7.3	66.8 ± 5.6	0.219	0.282
CO, L/min	5.0 ± 1.0	4.9 ± 1.2	5.2 ± 0.8	4.6 ± 1.0	0.073	0.312
LV mass, g	112.1 ± 19.5	110.7 ± 22.7	113.9 ± 15.2	105.7 ± 17.5	0.158	0.646
Right ventricle						
RVEDV, mL	130.3 ± 24.5	130.5 ± 25.7	130.0 ± 23.7	117.5 ± 26.3	0.041	0.954
RVESV, mL	64.3 ± 15.0	62.8 ± 12.9	65.5 ± 16.6	54.6 ± 13.2	0.006	0.610
RVSV, mL	66.0 ± 14.9	67.3 ± 18.1	65.0 ± 12.3	63.0 ± 18.7	0.458	0.686
RVEF, %	50.7 ± 6.8	51.3 ± 8.3	50.2 ± 5.4	52.9 ± 8.9	0.251	0.654

*BMI* body mass index; *SBP* systolic blood pressure; *DBP* diastolic blood pressure; *LVEDV* left ventricular end diastolic volume; *LVESV* left ventricular end systolic volume; *LVSV* left ventricular stroke volume; *LVEF* left ventricular ejection fraction; *CO* cardiac output; *LV mass* left ventricular mass; *RVEDV* right ventricular end diastolic volume; *RVESV* right ventricular end systolic volume; *RVSV* right ventricular stroke volume; *RVEF* right ventricular ejection fraction. ^a^Comparison between subjects with moderate to heavy alcohol consumption and healthy controls. ^b^Comparison between subjects with heavy and moderate alcohol consumption

### Comparison of CMR measurements

**Fig. 1:**
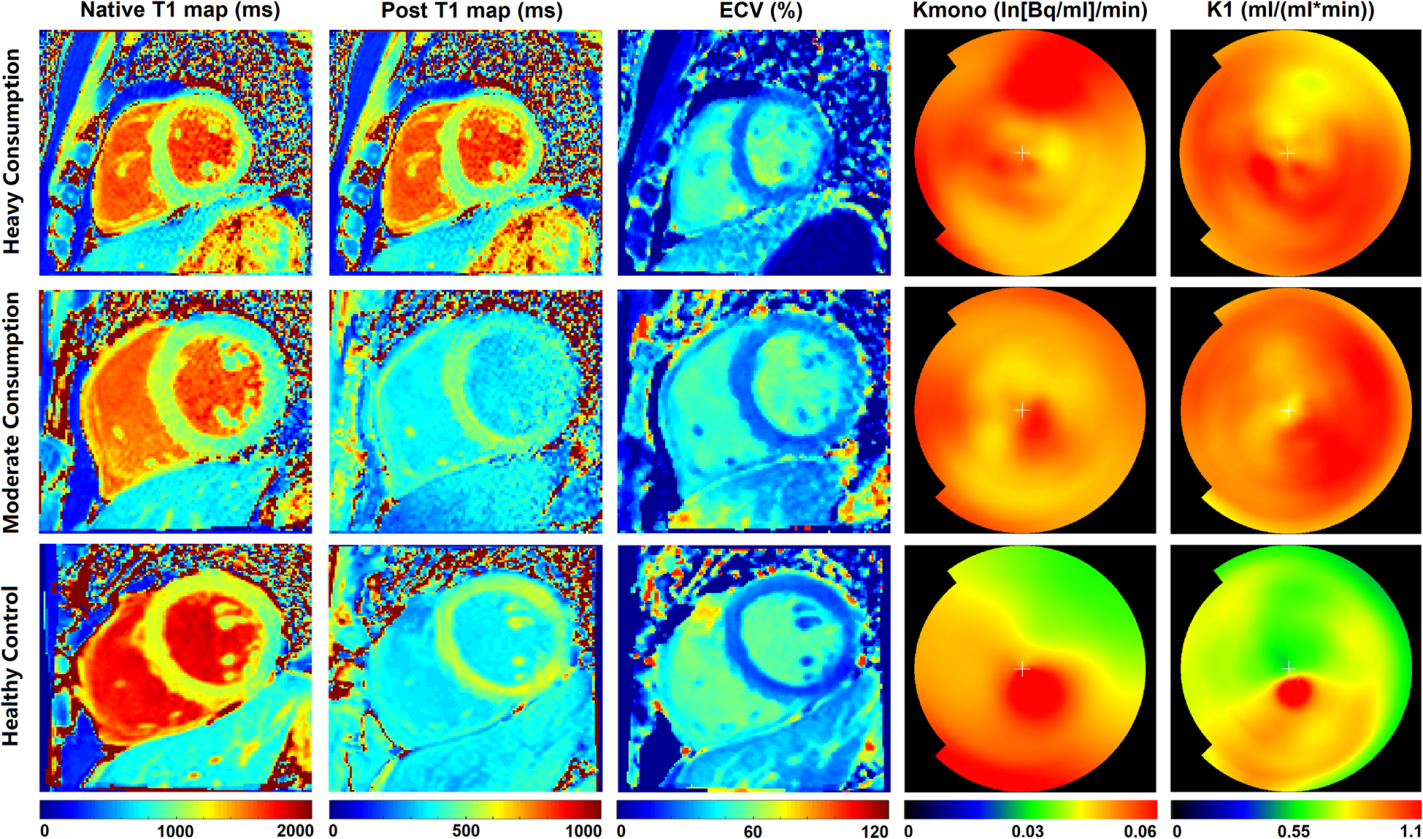
Example for comparison of imaging characteristics among subjects with heavy (age = 37 years) and (age = 39 years) moderate alcohol consumption, and healthy control (age = 40 years)

Table 2 summarizes the comparison results of the quantitative measurements from CMR and PET imaging among subjects with heavy to moderate alcohol consumption, and those without alcohol consumption. Compared to healthy controls, subjects with moderate to heavy alcohol consumption showed significantly shorter native and post T1 values, and greater ECV (all *p* < 0.05, Fig. 1). Significant differences were found in native T1 values (*p* = 0.004) between subjects with moderate alcohol consumption and healthy controls, but the differences in post T1 values and ECV were not statistically significant between these two groups (all *p* > 0.05). In contrast, subjects with heavy alcohol consumption showed significantly shorter native and post T1 values, and greater ECV than healthy controls (all *p* < 0.05). In addition, compared with subjects with moderate alcohol consumption, those with heavy alcohol consumption showed significantly shorter post T1 values (*p* = 0.036) and greater ECV (*p* = 0.034), but similar native T1 values (*p* = 0.791).

**Table 2:** Comparison between subjects with and without alcohol consumption

	Mean ± SD or n (%)				P^a^	P^b^	P^c^	P^d^
	Subjects with alcohol consumption			Healthy controls (*n* = 35)				
	All (*n* = 34)	Heavy consumption (*n* = 19)	Moderate consumption (*n* = 15)					
**CMR measurements**								
Native T1, ms	1133 ± 65	1130 ± 73	1136± 55	1186 ± 31	< 0.001	0.004	0.004	0.791
Post T1, ms	477 ± 42	464 ± 45	494 ± 32	501 ± 38	0.013	0.002	0.491	0.036
ECV, %	28.2 ± 2.2	28.9 ± 2.3	27.3 ± 1.9	26.9 ± 1.3	0.004	0.001	0.377	0.034
**PET measurements**								
Kmono, min^−1^ × 10^−3^	57.6 ± 12.1	52.9 ± 12.1	63.7 ± 9.2	63.0 ± 11.7||	0.189	0.028	0.861	0.007
K1, min^−1^	0.82 ± 0.13	0.80 ± 0.11	0.84 ± 0.16	0.83 ± 0.15||	0.833	0.589	0.865	0.444

*ECV* extracellular volume fraction. ^a^Comparison between subjects with moderate to heavy alcohol consumption and healthy controls. ^b^Comparison between subjects with heavy alcohol consumption and healthy controls. ^c^Comparison between subjects with moderate alcohol consumption and healthy controls. ^d^Comparison between subjects with heavy and moderate alcohol consumption. ||The measurements were taken from 12 healthy controls

Table 3 summarizes the results for the comparison of CMR and PET measurements using multivariate linear regression model by adjusting for confounding factors. The differences in CMR measurements of native T1, post T1 and ECV between subjects with moderate to heavy alcohol consumption and healthy controls remained statistically significant after adjusting for age, body mass index (BMI), history of smoking, and diastolic blood pressure (all *p* < 0.05). In contrast, no significant differences were found in all CMR measurements between subjects with heavy and moderate alcohol consumption after adjusting for age and systolic blood pressure (all *p* > 0.05, Fig. 1).

**Table 3:** Association between cardiac imaging measurements and alcohol consumption

	Alcohol consumption vs. healthy controls^a^			Heavy vs. moderate alcohol consumption^b^		
	β^c^	95% CI	P	β^c^	95% CI	P
**CMR measurements**						
Native T1, ms	− 59.19	−89, −30	< 0.001	− 8.83	−58, 40	0.714
Post T1, ms	−33.01	−57, −9	0.008	−21.16	−51, 8	0.155
ECV, %	1.63	0.58, 2.67	0.003	1.29	−0.25, 2.83	0.097
**PET measurements**						
Kmono, min^−1^ × 10^−3^	−10.67	−21.57, 0.24	0.055	−10.93	−19.26, −2.61	0.012
K1, min^−1^	−0.04	−0.17, 0.09	0.548	−0.01	− 0.12, 0.09	0.808

*ECV* extracellular volume fraction. ^a^The multivariate model is adjusted for age, BMI, history of smoke, and diastolic blood pressure. ^b^The multivariate model is adjusted for age, and systolic blood pressure. ^c^Values are the difference in MRI and PET imaging measurements between subjects with moderate to heavy alcohol consumption and healthy controls and between subjects with heavy and moderate alcohol consumption

**Table 4:** Association between CMR and PET measurements

MR measurements	PET measurements			
	Kmono		K1	
	r	P	r	P
Healthy controls				
Native T1	0.248	0.437	0.394	0.205
Post T1	−0.176	0.585	0.316	0.316
ECV	0.343	0.274	0.689	0.013
Moderate alcohol consumption				
Native T1	−0.011	0.970	0.057	0.840
Post T1	0.045	0.874	0.211	0.451
ECV	0.056	0.842	0.518	0.048
Heavy alcohol consumption				
Native T1	0.440	0.059	−0.263	0.276
Post T1	0.368	0.121	0.264	0.275
ECV	−0.207	0.395	−0.072	0.768

*ECV* extracellular volume

### Comparison of PET measurements

For the measurements from PET imaging, no significant differences in Kmono and K1 were observed between subjects with moderate to heavy alcohol consumption and healthy controls (all *p* > 0.05, Table 2). Similarly, the Kmono of subjects with moderate alcohol consumption didn’t significantly differ from those of healthy controls (*p* = 0.861). Meanwhile, subjects with heavy alcohol consumption showed significantly lower Kmono values compared with subjects with moderate alcohol consumption and healthy controls (all *p* < 0.05, Fig. 1). No differences in K1 were found among subjects with moderate and heavy alcohol consumption and healthy controls (all *p* > 0.05).

Multivariate linear regression analysis revealed that the differences in Kmono and K1 between subjects with alcohol consumption and healthy controls were not statistically significant after adjusting for confounding factors (all *p* > 0.05). In contrast, significant differences were found in Kmono (*p* = 0.012), but not in K1 (*p* = 0.808), between subjects with heavy and moderate alcohol consumption after adjusted for confounding factors (Table 3).

### Correlation between CMR and PET measurements

The correlations between CMR and PET measurements among different groups are shown in Table 4. Strong and moderate correlations were found between K1 and ECV in healthy controls (r = 0.689, *p* = 0.013) and subjects with moderate alcohol consumption (r = 0.518, *p* = 0.048), respectively. No significant correlations were found between K1, native, and post T1 values, or between Kmono, native, and post T1 values (all p > 0.05).

### Reproducibility of PET measurements

The inter-observer ICCs of Kmono and K1 were 0.997 (95%CI: 0.993–0.999) and 0.977 (95%CI: 0.944–0.991), respectively. The intra-observer ICCs of Kmono and K1 were 0.997 (95%CI: 0.987–0.999) and 0.977 (95%CI: 0.942–0.991), respectively.

## Discussion

This study investigated the characteristics of tissues and metabolism in myocardium of asymptomatic subjects with moderate to heavy alcohol consumption using quantitative CMR and PET/CT imaging. We found that compared to healthy controls, subjects with moderate to heavy alcohol consumption showed a significant decline in native T1 and post T1 values, and an increase in ECV. Subjects with heavy alcohol consumption showed similar native T1, post T1, and ECV values measured by CMR but had significantly lower Kmono measured by PET, compared to subjects with moderate alcohol consumption after adjusting for confounding factors. A linear correlation was found between ECV and K1 in healthy controls but this correlation was weakened as daily alcohol consumption increased.

In the present study, subjects with moderate to heavy alcohol consumption showed significantly shorter native and post T1 values compared with healthy controls. The decline of native T1 of myocardium may reflect myocardial fat deposition in individuals with alcohol consumption. da Silva et al. found that alcoholism was significantly associated with fat deposition in the LV myocardium (OR: 0.161; 95% CI: 0.072 to 0.36; *p* < 0.05) [16]. Experimental studies have demonstrated that chronic alcoholism stimulates the oxidative/nitrative stress, impairs myocardial mitochondrial function and fatty acid metabolism, and leads to steatosis and fat deposition in the myocardium [17].

The lack of significant differences in native and post T1 values between subjects with heavy and moderate alcohol consumption may be explained by the evidence that cardiac steatosis is an early phenomenon which is largely amplified by binges and prone to attenuation by increased duration of alcohol consumption [17]. In addition, focal replacement fibrosis of the myocardium in patients with alcoholic cardiomyopathy may attenuate the shortened T1 values by fat deposition [18]. Animal studies have shown that alcohol exposure modulates cardiac fibroblast matrix metalloproteinases (MMPs) and tissue inhibitors of MMPs (TIMPs) expression and stimulates TGF-β release from fibroblasts, favoring a profile associated with cardiac collagen accumulation [19, 20]. Though our study showed that there was a difference in native T1 between subjects with moderate to heavy alcohol consumption in comparison analysis, this difference was attenuated after adjusting for confounding factors. This phenomenon indicates that this difference may be influenced by age, BMI, and history of smoking and diastolic blood pressure. Our findings suggest that T1 measurements may not be a sensitive indicator for impairment of the myocardium in subjects with different doses of alcohol consumption.

We found that ECV of subjects with moderate to heavy alcohol consumption was significantly greater than that of healthy controls, but the differences between subjects with heavy and moderate alcohol consumption were not significant after adjusting for confounding factors. An increased ECV usually reflects a non-specific expansion of extracellular space or indicates myocardial fibrosis or an abnormal deposition of metabolites into the myocardium. Previous evidence indicates that chronic alcohol exposure gives cells a greater tendency to undergoing apoptosis via increasing the reactive oxygen species (ROS), which are crucial mediators in signal transduction during cell apoptosis process [21, 22]. The sequela of apoptosis, such as shrinkage and death of cardiomyocytes, may contribute to the increases in ECV in subjects with alcohol consumption. The mechanism for lack of differences in ECV between subjects with heavy and moderate alcohol consumption is unclear. As mentioned above, however, many pathophysiological changes are associated with the measurement of ECV.

In our study population, subjects with moderate alcohol consumption showed similar Kmono values to healthy controls, but this measurement was significantly reduced in those with heavy alcohol consumption. It has been shown that alcohol can inhibit mitochondrial respiration and the activity of enzymes in the tricarboxylic acid cycle, and interfere with both mitochondrial calcium uptake and binding synchronously [23]. The reduction of Kmono may therefore represent the inhibition of myocardial energy metabolism due to chronic alcohol consumption. Our subjects with moderate alcohol consumption having similar Kmono values to healthy controls may indicate compensatory sustaining of the myocardial metabolism, although there is apoptosis of cardiomyocytes caused by the toxicity of ethanol. Our findings suggest that Kmono measured by 11C-acetate PET/CT imaging might be an effective marker for the changes of myocardial metabolism in asymptomatic subjects with moderate to heavy alcohol consumption.

In the healthy controls and subjects with moderate alcohol consumption, our data showed that there was a significant correlation between ECV and K1. It is expected that K1 is positively correlated with ECV in the healthy controls. Although there was expansion of ECV in subjects with heavy alcohol consumption, K1 that represent myocardial perfusion were unchanged.

In the current study, some subjects with moderate to heavy alcohol consumption may be at the asymptomatic stage of alcoholic cardiomyopathy. Though their cardiac function is normal, these subjects may be at higher risk of progressing to symptomatic stages if abstinence or dose control are not applied. Compared with CMR imaging, 11C-acetate PET/CT imaging can detect metabolic disorders of myocardium in subjects with a long history of alcohol consumption. As such, multi-modal imaging may be an effective tool for stratifying the risk of cardiomyopathy caused by chronic alcohol consumption.

Our study has several limitations. First, this study only recruited asymptomatic subjects with moderate to heavy alcohol consumption. It will be valuable to determine the imaging features of symptomatic alcoholic cardiomyopathy patients. In addition, fat deposition within the myocardium might contribute to the decline of native T1 in subjects with alcohol consumption. However, fat quantification was not conducted in this study. Investigators utilized CMR techniques, such as fat-water separation imaging and H-CMR spectroscopy, to quantify cardiac fat deposition, and found that myocardial steatosis was a common phenomenon in idiopathic dilated cardiomyopathy and closely associated with myocardial triglyceride accumulation and diastolic dysfunction [24, 25]. Finally, only 1/3 of healthy subjects completed the PET imaging due to exposure to ionizing radiation.

## Conclusion

Asymptomatic men with moderate to heavy alcohol consumption have detectable changes in myocardial tissues and metabolism on quantitative CMR and 11C-acetate PET/CT imaging. Compared with quantitative CMR, 11C-acetate PET/CT imaging may be more sensitive for detecting differences in myocardial damage among subjects with moderate to heavy alcohol consumption.

## Data Availability

The CMR and 11C-acetate PET/CT data used and/or analyzed in the present study are available from the corresponding author on reasonable requests.

## Abbreviations

BMI: Body mass index
CMR: Cardiovascular magnetic resonance
CO: Cardiac output
ECM: Extracellular matrix
ECV: Extracellular volume
LV: Left ventricle/left ventricular
LVEDV: Left ventricular end diastolic volume
LVEF: Left ventricular ejection fraction
LVESV: Left ventricular end systolic volume
LVSV: Left ventricular stroke volume
MBF: Myocardial blood flow
MMPs: Matrix metalloproteinases
MVO_2_: Myocardial oxygen consumption
ROS: Reactive oxygen species
PET: Positron emission tomography
RVEDV: Right ventricular end diastolic volume
RVEF: Right ventricular ejection fraction
RVESV: Right ventricular end systolic volume
RVSV: Right ventricular stroke volume
TIMPs: Tissue inhibitors of MMPs
